# Overexpression of microRNA-205-5p exerts suppressive effects on stem cell drug resistance in gallbladder cancer by down-regulating PRKCE

**DOI:** 10.1042/BSR20194509

**Published:** 2020-09-29

**Authors:** Guo-Feng Zhang, Jia-Cheng Wu, Hong-Yong Wang, Wei-Dong Jiang, Ling Qiu

**Affiliations:** 1 Department of Hepatobiliary and Pancreatic Surgery, The Second Hospital of Jilin University, Changchun 130041, P.R. China; 2 Department of Radiation Oncology, The Second Hospital of Jilin University, Changchun 130041, P.R. China

**Keywords:** Apoptosis, Drug resistance, Gallbladder cancer, miR-205-5p, PRKCE, Proliferation

## Abstract

Some microRNAs (miRs or miRNAs) have been reported to function as tumor suppressors in gallbladder cancer (GBC). However, the specific effect of miR-205-5p on GBC remains unclear. The objective of the present study was to unravel the effects of miR-205-5p on the drug resistance in GBC. For this purpose, the expression of miR-205-5p and protein kinase C ϵ (PRKCE) was quantified in the peripheral blood sample harvested from GBC patients and healthy volunteers. Then the relationship between miR-205-5p and PRKCE was validated. After isolating the GBC stem cells, ectopic expression and depletion experiments were conducted to analyze the effect of miR-205-5p and PRKCE on cell proliferation, drug resistance, apoptosis, and colony formation rate as well as the expression of apoptotic factors (Bcl-2-associated X protein (Bax), B-cell lymphoma 2 (Bcl-2), and cleaved caspase 3). Finally, the mouse xenograft model of GBC was established to verify the function of miR-205-5p *in vivo*. Intriguingly, our results manifested that miR-205-5p was down-regulated, while PRKCE was up-regulated in peripheral blood samples and stem cells of patients with GBC. Moreover, miR-205-5p targeted PRKCE and negatively regulated its expression. The overexpression of miR-205-5p or silencing of PRKCE inhibited the drug resistance, proliferation, and colony formation rate while promoting apoptosis of GBC stem cells. Additionally, the overexpression of miR-205-5p attenuated drug resistance to gemcitabine but promoted the gemcitabine-induced cell apoptosis by inhibiting the PRKCE *in vivo*. Overall, an intimate correlation between miR-205-5p and PRKCE is a key determinant of drug resistance of GBC stem cells, thus, suggesting a novel miR-205-5p-based clinical intervention target for GBC patients.

## Introduction

Gallbladder cancer (GBC) is a major cause of cancer-related mortality in certain geographic areas over the world [1], mainly afflicting the female population with a gallstone in the geographic location such as northern India and Chile [2]. Importantly, it has been indicated that GBC remains asymptomatic in the early stages and often appears very late in the clinical course of the disease. Thus, the GBC is often diagnosed at its advanced stages [3]. Accordingly, in the advanced stage, the most common symptoms of patients with GBC are gallstones and chronic inflammation [4]. Though chemotherapy is a well-established strategy for cancer treatment. However, its effectiveness is significantly limited by tumor drug resistance [5]. Cancer cells usually possess multiple adaptive responses and mutations, like defective apoptotic mechanism, therefore, it is pivotal to strengthen the therapeutic efficiency and selectivity by overcoming drug resistance [6]. Therefore, it is considerably urgent to further explore the potential biomarkers that are significantly associated with drug resistance in GBC chemotherapy.

Protein kinase C ϵ (*PRKCE*) is a gene correlated to tumor aggressiveness and has been reported to be involved in malignant transformation and metastasis as it is up-regulated in various cancers, such as mammary and lung cancer [7]. Moreover, the oncogenic role of PRKCE in GBC has been elucidated, indicating its role in enhancing the gemcitabine resistance which can be attributed to the poor prognosis of GBC patients [8]. Besides, the PRKCE is capable of enhancing drug resistance by phosphorylating extracellular signal-regulated kinase (ERK), signal transducer and activator of transcription 3 (Stat3), activating transcription factor 2 (ATF2), phosphoinositide 3-kinase (PI3K), and P-glycoprotein (P-gp) [8]. The aberrations in microRNAs (miRNAs or miRs) have been frequently reported in GBC. Till date, the circulating levels of dozens of miRNAs were associated with GBC pathological characteristics [9]. Moreover, these miRNAs were revealed to target various tumor suppressors or oncogenes, by which these miRNAs play critical roles in GBC cell proliferation, apoptosis, invasion, and metabolism [9,10]. Recently, it was reported that PRKCE was a major target of several miRNAs, which were involved in tumorigenesis or drug resistance [11,12]. We speculated that *PRKCE* gene could also be modulated by miRNAs in GBC cells and aberrant miRNAs might disinhibit *PRKCE* gene to augment its oncogene-like functions. For example, the TargetScan website (http://www.targetscan.org/vert_71/) predicted the targeting relationship between the PRKCE and miR-205-5p. Of note, all genes in the human body are targeted by miRs which modulate the expression of their target genes and play a pivotal part in tumorigenesis. Particularly, the miR-205-5p has been proved to be a promising diagnostic biomarker of cancer owing to its capacity to decrease tumor chemoresistance and tumor progression [13]. A recently conducted research manifested that forced expression of miR-205-5p halts the growth of oral squamous cell carcinoma [14]. The miR-205-5p expression has indicated being down-regulated in diverse hepatocellular carcinoma (HCC) cell lines [15]. Additionally, the miR-205 enhances the sensitivity of pancreatic cancer cells to the gemcitabine and significantly reduces the proliferation of cancer stem cells and tumor growth in mouse models [16]. Therefore, it raised the possibility that miR-205-5p might target PRKCE and play a role in the resistance of GBC stem cells to gemcitabine. In the current study, we aimed to identify potential miRNAs that could regulate *PRKCE* gene by comparing with the prediction results from three distinct online tools. The functional interaction between PRKCE and the most promising miRNAs was then investigated.

## Materials and methods

### Study subjects

From March 2015 to June 2018, 68 patients (26 males and 42 females, aged 43–72 years with an average age of 56.1 ± 4.9 years) pathologically diagnosed with GBC and underwent surgery at The Second Hospital of Jilin University, were selected as study subjects. According to the sixth edition of the American Joint Committee on Cancer (AJCC) tumor-node-metastasis (TNM) system, 25 cases were at stage III, 20 cases at stage IV A, and 23 cases at stage IV B. The main clinical symptoms were as follows: 39 cases with abdominal distension and pain, 20 cases with jaundice, 7 cases with an abdominal mass, 1 case with progressive emaciation, and 1 case with melena. All patients had not received any preoperative treatments 1 month before the enrollment, including radiotherapy or chemotherapy, or medicines which would affect their immune system. Patients had normal liver and kidney function and were free from a history of other malignancies. Then, 68 healthy volunteers including 30 males and 38 females (aged 40–70 years with an average age of 52.4 ± 4.3 years) receiving health examination during the same period were selected as controls. There was no statistical difference in age, course of the disease, and other general data between the two groups (*P*>0.05).

### Reverse transcription-quantitative polymerase chain reaction

Ten milliliters of peripheral venous blood samples were harvested from the GBC patients and healthy volunteers under empty stomach condition in the morning followed by the extraction of total RNA. Then, the extracted RNA was reverse transcribed into complementary DNA (cDNA) using TaqMan miRNA reagent kit (Applied Biosystems, Foster City, CA, U.S.A.). Primer Premier 5.0 was applied to design the primers for reverse transcription-quantitative polymerase chain reaction (RT-qPCR; Table 1). The RT-qPCR protocol was set at 95°C for 2 min followed by 40 cycles at 95°C for 15 s, at 68°C for 45 s, and at 72°C for 15 s. The relative expression of PRKCE or miR-205-5p was normalized to glyceraldehyde-3-phosphate dehydrogenase (GAPDH) or U6 and assessed using the 2^−ΔΔ*C*_t_^ method. This assay was also applicable in cells after 48 h of transfection.

**Table 1:** Primer sequences for RT-qPCR

Gene	Primer sequence
*miR-205-5p*	F: 5′-TCCACCGGAGTCTGTCTCAT-3′
	R: 5′-GCTGTCAACGATACGCTACG-3′
*PRKCE*	F: 5′-AGTACGGCCCCTCAGTGGA-3′
	R: 5′-ATCGTCCTCGTTGTCAGCCTC-3′
*BRCA1*	F: 5′-AATATTTGGGAAAACCTATCGGA-3′
	R: 5′-GGGACGCTCTTGTATTATCTGTG-3′
*U6*	F: 5′-CTCGCTTCGGCAGCACA-3′
	R: 5′-AACGCTTCACGAATTTGCGT-3′
*GAPDH*	F: 5′-ATGGTGAAGGTCGGTGTGAA-3′
	R: 5′-GAGTGGAGTCATACTGGAAC-3′

Abbreviations: BRCA1, BRCA1 DNA repair associated; F, forward.

### Isolation and identification of GBC stem cells

The single cell suspension was prepared by dispersing human GBC cells GBC-SD (Shanghai Institutes for Biological Sciences, Chinese Academy of Sciences, Shanghai, China) and then supplemented into serum-free Dulbecco’s modified Eagle’s medium/Ham’s F-12 medium (DMEM/F12; Gibco, Carlsbad, CA, U.S.A.) containing multiple cytokines for tumor cell clone development. Then, flow cytometry was conducted to identify the expression of cancer stem cell markers, CD44 and CD133, in cell colonies and adherent cells. After the cell density was adjusted to 1 × 10^7^ cells/ml, the cell suspension was packed in 5-ml flow tubes (2 ml/tube) followed by culture with human immunoglobulin M solution, anti-CD44-allophycocyanin (Becton, Dickinson, and Company, Franklin Lake, NJ, U.S.A.), and anti-CD133/1-phycoerythrin (eBioscience company, Ben Lomond, CA, U.S.A.), respectively. After incubation, the expression of CD44 and CD133 was detected on a flow cytometer with the primary antibodies, including mouse anti-human CD44 antibody (Wuhan Boster Biological Technology, Ltd., Wuhan, Hubei, China) and mouse anti-human CD133 antibody (Abcam, Cambridge, U.K.). CD44^+^, CD44^−^, CD133^+^, and CD133^−^ were isolated and the cells were routinely sorted twice to enable cell purity to reach over 90%.

### Luciferase reporter assay

The biological prediction website, TargetScan (http://www.targetscan.org/vert_71/) was used to analyze the target gene of miR-205-5p. According to the instructions of the Omega plasmid extraction kit (D6943-01, Zhijiefangyuan Technology Co., Ltd., Beijing, China), plasmids were extracted to construct the recombinant plasmids, i.e., pmirGLO-PRKCE-3′untranslated region (UTR) wildtype (WT) and pmirGLO-PRKCE-3′UTR mutant type (MUT). The correctly sequenced luciferase reporter plasmids PRKCE-WT and PRKCE-MUT were respectively co-transfected with miR-205-5p mimic or NC mimic into HEK-293T cells (CRL-1415, Shanghai Xin Yu Biotech Co., Ltd., Shanghai, China) using a luciferase assay kit (RG006, Beyotime Institute of Biotechnology, Shanghai, China). The relative luciferase (RLU) activity equaled *Renilla* luciferase activity (pRL-TK) divided by the firefly luciferase activity. The RLU activity was calculated with the cell lysate reporter gene as the blank control.

### Cell transfection

Mimic-negative control (NC), miR-205-5p mimic, inhibitor-NC, miR-205-5p inhibitor, small interfering RNA (si)-NC, si-PRKCE#1, and si-PRKCE#2 plasmids were bought from the GenePharma (Shanghai, China). Based on the manufacturer’s manuals, the Lipofectamine 2000 (Invitrogen, Carlsbad, CA, U.S.A.) was adopted to transfect CD44^+^ CD133^+^ GBC-SD cells.

### Colony formation assay

The CD44^+^ CD133^+^ GBC-SD cells in the exponential growth phase were dispersed into cell suspension. Afterward, 100-μl cell suspension was cultured in a 96-well plate for 14 days until the cell colonies were observed with naked eyes. After 15 min of fixation with 4% paraformaldehyde, cell colonies were stained with 0.1% Crystal Violet. The colonies (with more than twn cells) were counted with naked eyes with a transparent film and observed under a microscope (CX21; Olympus Optical Co., Ltd., Tokyo, Japan). Finally, the colony formation rate was calculated as the number of colonies formed/number of inoculated cells × 100%.

### Cell counting kit-8 assay

The cell counting kit-8 (CCK-8) reagent kit (96992-500TESTS-F, Sigma–Aldrich, St. Louis, MO, U.S.A.) was used to test the GBC-SD viability. Transfected cells were resuspended in DMEM and inoculated in a 96-well plate at a density of 4000 cells/well (100 ml/well) at 37°C with 5% CO_2_. After adhering to the wells, the cells were divided into eight groups with one blank control (no treatment) set. After the treatment, CCK-8 (10 ml) was supplemented to each well at an interval of 2 h. A microplate reader (Bio-Tek, Norcross, GA, U.S.A.) was applied to evaluate optical density (OD) value at 450 nm. Similarly, gemcitabine at different concentrations (0, 10^−3^, 10^−2^, 10^−1^, 10^0^, 10^1^, 10^2^, and 10^3^ μM) was used to observe cell cytotoxicity.

### Flow cytometry

The cell apoptosis was analyzed according to the manuals of Annexin V-fluorescein isothiocyanate (FITC)/propidium iodide (PI) reagent kit (Dickinson and Company). After 48 h of transfection, cells were collected. Then, approximately 1 × 10^5^ cells were resuspended in 500 ml binding buffer and reacted with the mixture of 5 ml Annexin V-FITC and 5 ml PI solution in the dark at ambient temperature for 15 min, respectively. The samples were analyzed on a BDFACSCantoII flow cytometer within 1 h with the CellQuest software (Becton, Dickinson, and Company). The test was repeated three times.

### Western blot analysis

The total protein was extracted with radio-immunoprecipitation assay (RIPA) cell lysis buffer from cells 48 h after transfection. Following the application of 8% polyacrylamide gel electrophoresis, the protein samples were electroblotted to a polyvinylidene fluoride membrane (Millipore, Billerica, MA, U.S.A.). After 1 h of blocking with 5% dried skimmed milk at ambient temperature, the membrane was probed with primary rabbit antibodies to PRKCE (1:2000, ab181558), Bcl-2-associated X protein (Bax) (1:2000, ab32503), B-cell lymphoma 2 (Bcl-2) (1:1000, ab196495), cleaved caspase3(3 1:500, ab49822), and β-actin (1:5000, ab179467) overnight at 4°C. Then, the membrane was re-probed with horseradish peroxidase (HRP)-conjugated secondary antibodies for 1 h at 37°C in a shaker. The immunocomplexes on the membrane were visualized using enhanced chemiluminescence and band intensities were quantified using the Image 7.0 system (National Institutes of Health, Bethesda, MD, U.S.A.). The relative protein expression was expressed by the gray value of the target protein band to that of the β-actin protein band.

### Xenograft tumor in nude mice

Forty male BALB/C nude mice (Beijing Vital River Laboratory Animal Technology Co., Ltd., Beijing, China; aged 4–6 weeks; weighing 18–20 g) were selected for the present study. Stably transfected cells with mimic-NC and miR-205-5p mimic were dispersed into cell suspensions (2 × 10^6^ cells/ml) with normal saline. Finally, 0.2 ml cell suspension was subcutaneously injected into the lateral femur of each mouse with a 1-ml injector. One week later, mice in different groups underwent intraperitoneal injection of gemcitabine (15 mg/kg) or saline (100 μl; NC) weekly. Vernier caliper was used to observe the tumor growth of nude mice once a week, for a total of 5 weeks. The volume of tumors was calculated as follows: V = 1/2 × ab^2^, in which ‘a’ referred to tumor length and ‘b’ referred to tumor width. All mice were anesthetized with ≥100 mg/kg sodium pentobarbital via intraperitoneal injection and then killed by cervical dislocation at the fifth week, the tumors were dissected out and weighed for immunohistochemistry and terminal deoxynucleotidyl transferase dUTP nick end-labeling (TUNEL) staining, respectively. The animal work took place at The Second Hospital of Jilin University.

### Immunohistochemistry

After conventional dehydration, 4-μm-thick paraffin slices of tumor tissues were made. After slice dewaxing and hydration, the citric acid buffer was used for antigen retrieval at high temperatures. Then, the endogenous peroxidase activity was blocked with 30 ml/l H_2_O_2_. Slices were then probed with the primary antibody, polyclonal rabbit antibody to PRKCE (1:100; ab23511, Abcam) at 4°C overnight and re-probed with biotinylated HRP-conjugated secondary goat-anti-rabbit (1:100; Zhongshan Bio-Tech Co., Ltd., Zhongshan, Guangdong, China) at 37°C for 30 min. The slices were reacted with HRP and developed with diaminobenzidine (DAB; Maixin Bioengineering Company, Fuzhou, Fujian, China). Following Hematoxylin counterstaining, routine dehydration, clearing, and mounting followed by observation of the slices under an optical microscope (XSP-36, Boshida Optical Instruments Co., Ltd., Shenzhen, Guangdong, China). The positive cells in 1000 cells were calculated while the positive rate = (number of positive cells/total number of cells) × 100%.

### TUNEL staining

The tumor tissues were conventionally dehydrated, embedded in paraffin, and finally cut into 4-μm sections. Proteinase K (20 mg/ml) was dissolved into 10 mM Tris-HCl (pH = 7.4–8.0). After hydration for 15 min, the sections were incubated with 20 μl TUNEL reaction mixture in the wet box at 37°C for 60 min. Afterward, the sections were developed with a 50-μl DAB solution (Maixin Bioengineering Company, Fuzhou, Fujian, China) under a microscope, then counterstained with Hematoxylin, and mounted. Positive control and NC were set at the same time. Brown-yellow granules appearing in the nucleus served as a positive control. The number of TUNEL-positive cells was randomly counted in five fields and the apoptosis index (AI) for each field was calculated as the percentage of TUNEL-positive cells relative to the total cells.

### RNA pull-down assay

The HEK293T cells were cultured in 90-mm cell culture dishes. For cells harvest, cells were first rinsed with phosphate-buffered saline (PBS) and collected in a 1.5-ml test tube using a cell scraper. Then, the tube was added with 0.5 ml of 25 mM Tris-HCl (pH 7.5), 70 mM KCl, 2.5 mM ethylenediaminetetraacetic acid (EDTA), 0.5% NP-40, 80 U/ml RNase inhibitor (Shengong, China), 1× protease inhibitor cocktail (Sigma, U.S.A.), respectively, and incubated on ice for 20 min until the cells were completely lysed. The cell lysate was centrifuged at 12000×***g*** and 4°C for 15 min and the supernatant was transferred to a new tube. Then, 500 μl supernatant was added with biotinylated double-stranded RNA (8 nmoles), miR-205-5p (Sense: 5′-p-UCCUUCAUUCCACCGGAGUCUG-biotin-3′, Antisense: 5′-p-GAUUUCAGUGGAGUGAAGUUC-biotin-3′) or control random RNA (Sense: 5′-p-AUCCGCGCGAUAGUACGUAUU-biotin-3′, Antisense: 5′-p- UACGUACUAUCGCGCGGAUUU-biotin-3′), and then incubated at 4°C for 30 min. Thereafter, another 1 h of incubation was conducted at 30°C. The products were added to 10 μl Streptavidin Mutein Matrix (Roche Applied Science, U.S.A.) and incubated at 4°C for 1 h followed by two washes using extraction buffer (250 μg RNase-free BSA and 100 μg yeast tRNA in 500 μl of 25 mM Tris-HCl [pH 7.5], 70 mM KCl, 2.5 mM EDTA, and 0.5% NP-40). After centrifugation at 5000×***g*** and 4°C for 30 s, Streptavidin/biotin–miRNA/mRNA complex was collected and washed five times (5 min/time) using 20 mM Tris-HCl (pH 7.4), 400 mM KCl, and 0.5% NP-40 at 4°C. Then, the biotin–miRNA/RNA complex was eluted with 250 μl of 20 mM Tris-HCl (pH 7.4), 400 mM KCl, 0.5% NP-40, 5 mM biotin, and 80 U/ml RNase inhibitor at 42°C for 5 min. The products were treated with DNase-I at 37°C for 10 min, then extracted and purified with phenol–chloroform, followed by reverse transcription [17]. The purified RNA was then detected by RT-qPCR.

### Statistical analysis

All data were summarized as the mean ± standard deviation and analyzed by the SPSS 21.0 software (IBM, Armonk, NY, U.S.A.), with a value of *P*<0.05 representing statistical significance. The normally distributed data among multiple groups were evaluated using the Kolmogorov–Smirnov test. Student’s *t* test was applied for pairwise comparison. Kruskal–Wallis (non-parametric) test was used for data with skewed distribution. The relationship between miR-205-5p and PRKCE expression and the overall survival (OS) of GBC patients was determined using the Kaplan–Meier method and compared by the log-rank test.

## Results

### Bioinformatics analysis predicting that miR-205-5p regulates PRKCE to affect drug resistance of GBC stem cells

The PRKCE has reported being related to drug resistance [18] and poor prognosis [8]. However, the mechanism of PRKCE regulating drug resistance of GBC stem cells has rarely been studied. In the present study, we first investigated the expression of PRKCE in GBC. The GEPIA database (http://gepia.cancer-pku.cn/) [19] predicted that PRKCE was highly expressed in the GBC (Supplementary Figure S1A). Then, the miRNAs potentially targeting PRKCE were predicted using the TargetScan (http://www.targetscan.org/vert_71/), MicroSearch V3.0 (https://www.exiqon.com/mirsearch) and starBasev2.0 (http://starbase.sysu.edu.cn) websites, and the results were subsequently intersected (Supplementary Figure S1B), with six potential miRNAs obtained (hsa-miR-205-5p, hsa-miR-96-5p, hsa-miR-31-5p, hsa-miR-103a-3p, hsa-miR-1271-5p, and hsa-miR-107). Furthermore, we analyzed the relationship between the six miRNAs and drug resistance using the CCK-8 assay. Our results showed that miR-205-5p was related to drug resistance (Supplementary Figure S1C). Therefore, miR-205-5p was selected as the regulatory miRNAs of PRKCE for subsequent studies.

### miR-205-5p is down-regulated and PRKCE is up-regulated in peripheral blood samples of patients with GBC

**Figure 1:**
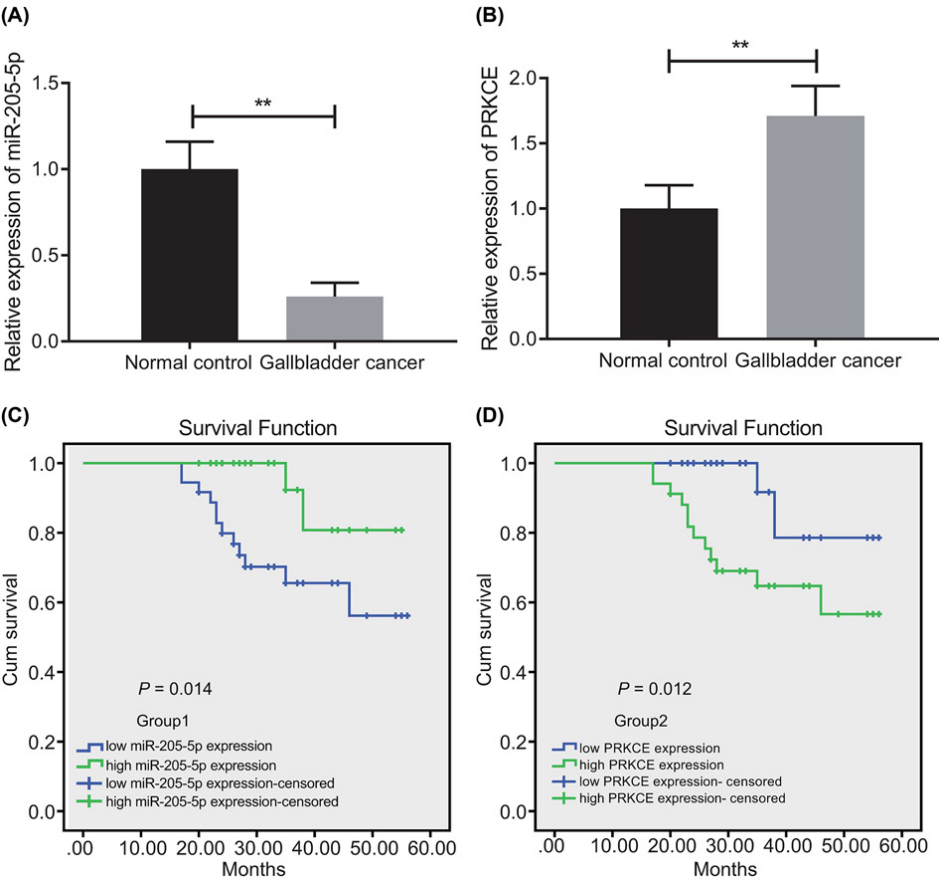
Low miR-205-5p expression and high PRKCE expression are observed in peripheral blood samples of patients with GBC (**A**) The expression of miR-205-5p in peripheral blood samples of patients with GBC and healthy controls measured by RT-qPCR. (**B**) The mRNA expression of PRKCE in peripheral blood samples of patients with GBC and healthy controls assessed by RT-qPCR. (**C**) The analysis of the OS of patients with different expressions of miR-205-5p by Kaplan–Meier. (**D**) The analysis of the OS of patients with different PRKCE mRNA expression by Kaplan–Meier. ‘Expression-censored’ represented patients who could not be contacted or were no longer willing to be followed up during our later follow-up. The method of gene expression grouping is as follows: first, the relative expression of miR-205-5p or PRKCE normalized to the internal reference gene in all patient samples is counted, and then the median of relative expression in all patients is taken. High expression is higher than the median, and low expression is lower than the median. *n*=68. **P<0.01. The basis of high and low expression is the median.

RT-qPCR was performed to observe the difference of miR-205-5p and PRKCE expression in GBC patient samples, which described that in contrast with healthy control samples, miR-205-5p expression was down-regulated while PRKCE mRNA expression was up-regulated in the peripheral blood sample of GBC patients (Figure 1A,B). In order to understand the relationship between the expression of miR-205-5p and the survival time of patients, patients were divided into high and low expression according to the median value of miR-205-5p and PRKCE expression. Further analysis of the Kaplan–Meier method depicted that the OS of GBC patients with high expression of miR-205-5p was higher, whereas the expression of GBC patients with low expression of miR-205-5p was lower. Moreover, the correlation between the OS of patients and PRKCE expression was negative (Figure 1C,D). These results demonstrated that miR-205-5p was poorly expressed and PRKCE was highly expressed in the peripheral blood samples of patients with GBC. However, low expression of miR-205-5p and high expression of PRKCE were indicative of poor prognosis.

### miR-205-5p is down-regulated and PRKCE is up-regulated in GBC stem cells

**Figure 2:**
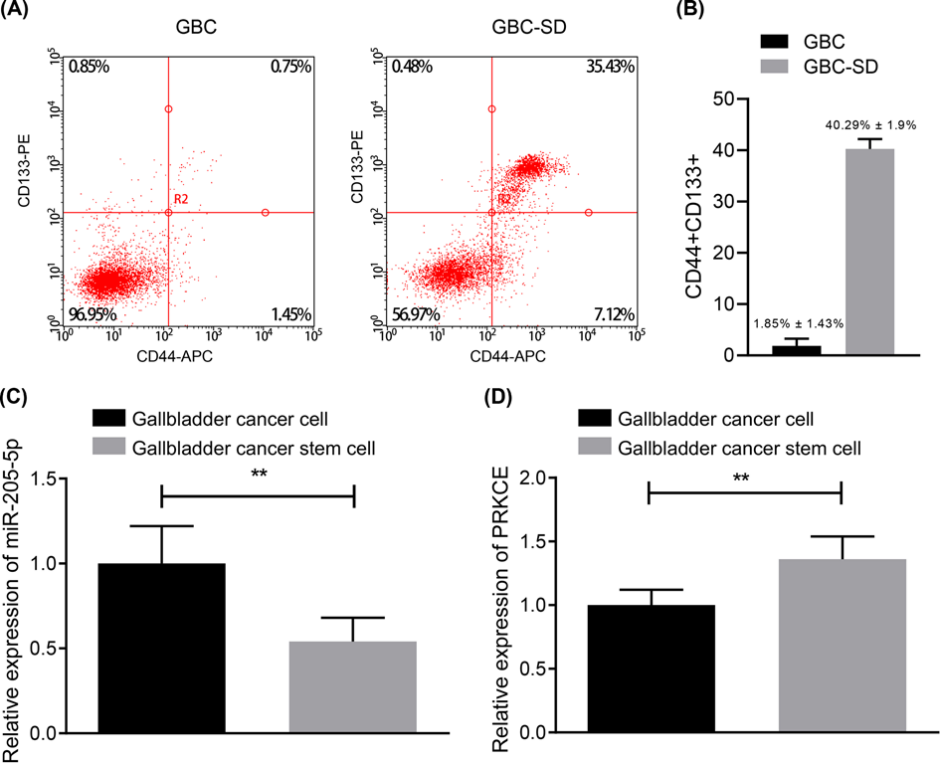
Low expression of miR-205-5p and high expression of PRKCE in GBC stem cells (**A**) The expression of cell markers including CD44 and CD133 in primary GBC cells analyzed by flow cytometry. (**B**) The expression of cell markers including CD44 and CD133 in GBC-SD cells determined by flow cytometry. (**C**) The expression of miR-205-5p in GBC cells and GBC stem cells by RT-qPCR. (**D**) The mRNA expression of PRKCE in GBC cells and GBC stem cells evaluated by RT-qPCR **P<0.01. The above results were all measurement data, expressed as the mean ± standard deviation and tested by unpaired *t* test. The experiment was repeated three times independently.

The above-mentioned findings revealed reduced miR-205-5p and amplified PRKCE in clinical samples as well as their significant correlation with patient survival. We, therefore, speculated that the PRKCE and miR-205-5p may function in GBC through tumor stem cells [20]. To understand the relationship between PRKCE, miR-205-5p and tumor stem cells, flow cytometry was performed to sort GBC stem cells from clinical samples. CD44^+^ CD133^+^ GBC stem cells accounted for 1.85 ± 1.43% in primary GBC cells and for 40.29 ± 1.9% in GBC-SD cells (Figure 2A). Then, RT-qPCR was conducted to measure the miR-205-5p expression and PRKCE mRNA expression in GBC stem cells, which exhibited the reduced expression of miR-205-5p (Figure 2B) and highly expressed PRKCE mRNA in GBC stem cells as compared with the GBC cells (Figure 2C,D). Conclusively, our results revealed that in GBC stem cells down-regulated the miR-205-5p while up-regulated the PRKCE.

### miR-205-5p negatively regulates PRKCE

**Figure 3:**
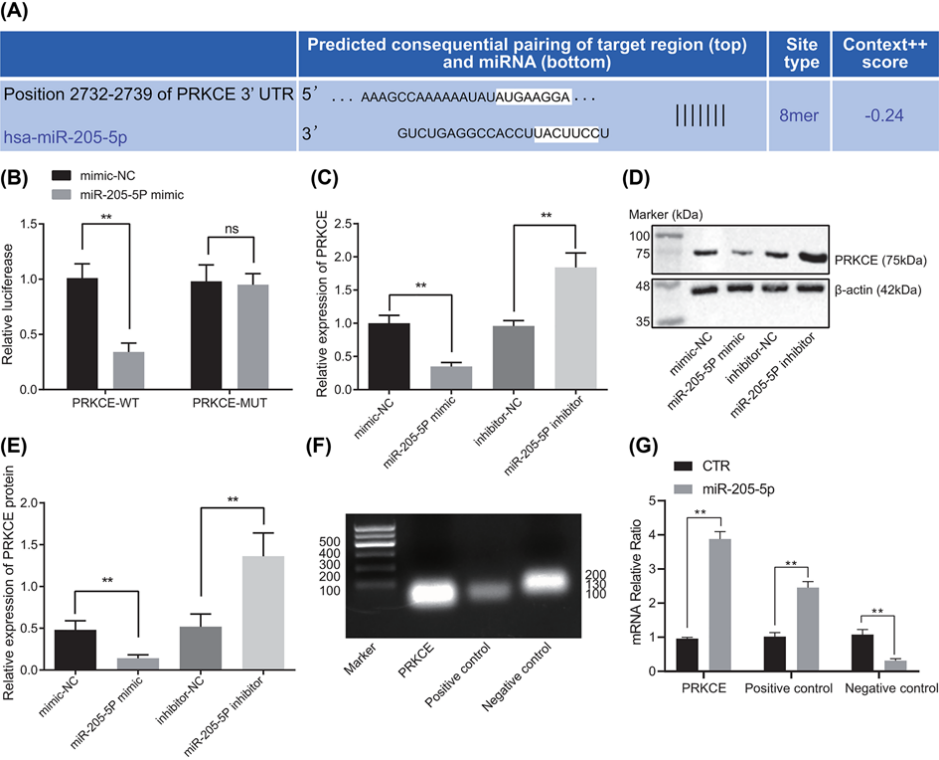
*PRKCE* is a target gene of miR-205-5p (**A**) Predicted binding sites between miR-205-5p and PRKCE by the TargetScan database. (**B**) The binding of miR-205-5p to PRKCE confirmed by dual-luciferase reporter gene assay. (**C**) The mRNA expression of PRKCE in cells after the alteration of miR-205-5p examined by RT-qPCR. (**D**) The protein expression of PRKCE in cells after the alteration of miR-205-5p determined by Western analysis. (**E**) Statistical results of (D). (**F**) Genes to be measured by RNA pull-down assay were all detected in HEK293 cells. BRCA1 mRNA known to be regulated by miR-205-5p was used as the positive control and GAPDH mRNA was used as the NC. The sizes of DNA ladders and actual sizes of PCR products were demonstrated on the left and right sides of the figure, respectively. (**G**) The mRNA levels of PRKCE, BRCA1 (positive control), and GAPDH (NC) in biotinylated miRNA pull-down products examined by RT-qPCR. CTR indicates biotinylated random miRNA. **P<0.01. The above results were all measurement data, expressed as the mean ± standard deviation and tested by unpaired *t* test. The experiment was repeated three times independently.

Following the identification of high expression of PRKCE and low expression miR-205-5p in GBC stem cells, we attempted to further analyze their correlation. The TargetScan website (http://www.targetscan.org/vert_71/) predicted PRKCE as a target gene of miR-205-5p (Figure 3A). To verify the predicted results, dual-luciferase reporter assay was used and our results indicated that the luciferase activity was significantly decreased in PRKCE-WT and miR-205-5p mimic co-transfected cells (*P*<0.01). However, no changes were observed in the luciferase activity of the cells co-transfected with PRKCE-MUT and miR-205-5p mimic (*P*>0.05) (Figure 3B), indicating that miR-205-5p can specifically target PRKCE.

Furthermore, RT-qPCR and Western blot analysis manifested strikingly reduced expression of PRKCE following miR-205-5p mimic treatment whereas the expression of PRKCE was remarkably enhanced when treated with the miR-205-5p inhibitor (Figure 3C–E). Further dual-luciferase reporter assay results indicated that miR-205-5p can specifically target PRKCE. The RNA pull-down assay was therefore performed with biotinylated random miRNA control (CTR) or biotinylated miR-205-5p to confirm the physical interaction between miR-205-5p and PRKCE. We used BRCA1 mRNA known to bind to miR-205-5p as a positive control and GAPDH mRNA as a NC (Figure 3F). RT-PCR confirmed the expressions of PRKCE, BRCA1, and GAPDH in HEK293T cells. Analysis of precipitated miRNA–mRNA complex demonstrated that more PRKCE and BRCA1 (positive control), but not GAPDH (NC) mRNA was enriched in the miR-205-5p group than in the CTR group (Figure 3G). These results indicated that miR-205-5p could directly bind to PRKCE and negatively regulate its protein expression *in vitro*.

### Overexpression of miR-205-5p or silencing of PRKCE inhibits the drug resistance and promotes apoptosis of GBC stem cells

**Figure 4:**
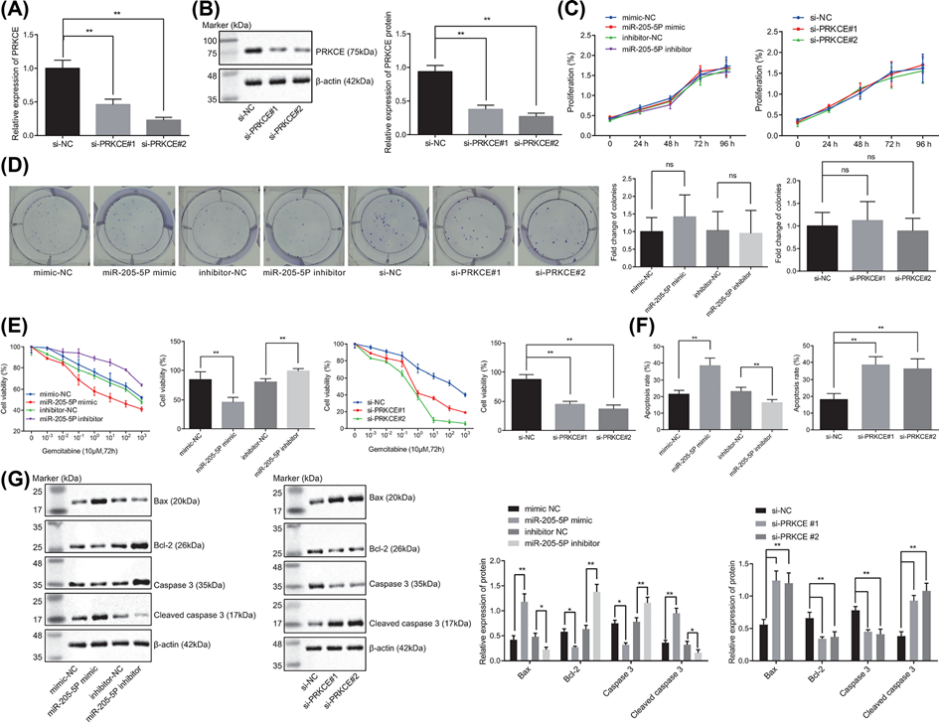
Overexpressing miR-205-5p or silencing PRKCE represses the drug resistance and induces apoptosis in GBC stem cells (**A**) The silencing efficiency of PRKCE in cells determined by RT-qPCR. GBC stem cells were transfected with mimic-NC, miR-205-5p mimic, inhibitor-NC, miR-205-5p inhibitor, si-NC, si-PRKCE#1 or si-PRKCE#2. (**B**) Protein expression of PRKCE in cells determined by Western blot analysis. (**C**) Cell proliferation determined by CCK-8 assay. (**D**) Colony formation determined by colony formation assay. (**E**) The drug resistance of cells determined by CCK-8 assay (concentration of gemcitabine: 0.1 M). (**F**) Cell apoptosis determined by Annexin V/PI. (**G**) The protein expression of apoptosis-related genes in cells determined by Western blot analysis. *P<0.05, **P<0.01. The above results were all measurement data, expressed as mean ± standard deviation. One-way analysis of variance was used for multigroup comparisons, while repeated-measures analysis of variance was applied for data comparison at different time points. The experiment was repeated three times independently.

To study the effects of miR-205-5p or PRKCE on drug resistance of GBC stem cells, a series of assays were performed including RT-qPCR, Western blot analysis, colony formation assay, CCK-8 assay, and Annexin V/PI. The results of RT-qPCR and Western blot analysis (Figure 4A,B) presented that PRKCE expression was substantially diminished after the treatment of si-PRKCE #1 or si-PRKCE #2. The colony formation and CCK-8 assays demonstrated no prominent difference regarding cell proliferation (Figure 4C) and colony formation (Figure 4D) after various treatments (*P*>0.05). Gemcitabine was used for drug resistance testing and the experimental results showed that miR-205-5p mimic or silencing PRKCE resulted in diminished drug resistance while miR-205-5p inhibitor enhanced drug resistance (Figure 4E). Furthermore, miR-205-5p mimic or silencing the PRKCE contributed to a noteworthy elevation in cell apoptosis and the expression of pro-apoptotic factors (Bax and cleaved caspase 3) yet a decline in the expression anti-apoptotic factor (Bcl-2) was observed. On the contrary, the miR-205-5p inhibitor led to opposite results (Figure 4F,G). These aforementioned results supported that up-regulated miR-205-5p or silencing PRKCE might decrease drug resistance and promote the apoptosis of GBC stem cells.

### Overexpressed miR-205-5p inhibits PRKCE expression and the growth of gemcitabine-resistant cells *in vivo*

**Figure 5:**
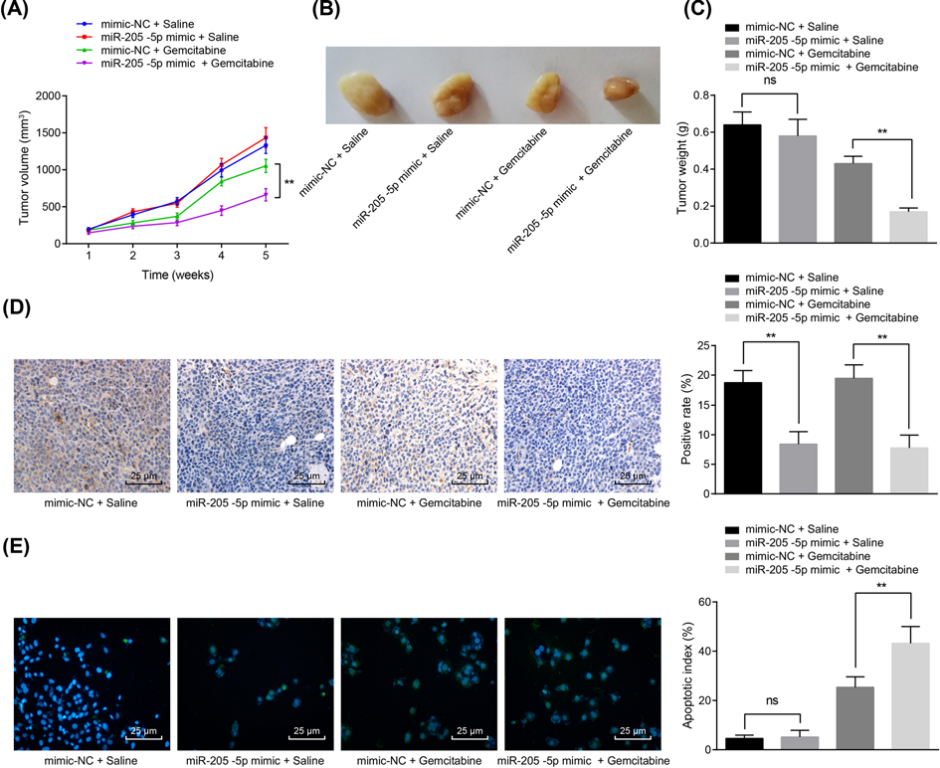
Overexpression of miR-205-5p represses PRKCE expression and the gemcitabine resistance of GBC cells *in vivo* Mice were treated with mimic-NC + saline, miR-205-5p mimic + saline, mimic-NC + gemcitabine or miR-205-5p mimic + gemcitabine. (**A**) Tumor volume of nude mice. (**B**) Representative tumor images of nude mice. (**C**) Tumor weight in the fifth week in nude mice. (**D**) The expression of PRKCE determined by immunohistochemistry (×400, scale bar = 25 μm). (**E**) Tumor tissue apoptosis in nude mice measured by TUNEL staining (×400, scale bar = 25 μm). *n*=10. **P<0.01. The above results were all measurement data, expressed as mean ± standard deviation. One-way analysis of variance was used for multigroup comparisons, while repeated-measures analysis of variance was applied for data comparison at different time points.

To further understand the role of miR-205-5p in inhibiting PRKCE expression and tumor growth, tumor formation assay, immunohistochemistry, and TUNEL staining were performed in nude mice. There was no distinct change in tumor volume and weight between mice injected with saline in the presence of mimic-NC or miR-205-5p (*P*>0.05). In mice injected with gemcitabine, the volume and weight of tumors were reduced by miR-205-5p mimic (Figure 5A–C). The results of immunohistochemistry showed that in the presence of saline or gemcitabine, the expression of PRKCE was reduced in mice when treated with the miR-205-5p mimic (Figure 5D). Additionally, in the presence of gemcitabine, the apoptosis rate was enhanced in mice treated with miR-205-5p mimic (Figure 5E). Collectively, miR-205-5p negatively regulated the PRKCE and enhanced gemcitabine-induced apoptosis and tumor growth *in vivo*.

## Discussion

GBC is a relatively rare neoplasm and considered to be a lethal disease with high mortality [21]. Though specific miRNAs have been reported to participate in the GBC development by regulating cell proliferation, metastasis, invasion, and apoptosis [22]. However, the role of miR-205-5p as an oncogenic or tumor-suppressing factor in GBC remained unclear. Accordingly, the present study was intended to identify the novel targets regulated by miR-205-5p in GBC and to unravel its role in the molecular mechanism of GBC. Notably, our study found that miR-205-5p repressed the drug resistance of GBC stem cells to the gemcitabine by targeting the PRKCE.

The most important finding of our study was the poor expression of miR-205-5p while high expression of the PRKCE in peripheral blood samples and GBC stem cells in patients with GBC. Consistently, a recent study has found the up-regulated PRKCE in triple-negative breast cancers [23]. Moreover, PRKCE has also reported being abundantly expressed in papillary thyroid carcinoma [24]. On the other hand, miRNAs have manifested being involved in cellular differentiation, migration, and proliferation [20]. Particularly, in breast cancer, the low expression of miR-205-5p has been attributed to the poor prognosis, which further contributed to breast cancer progression to metastasis [25]. Another research documented that the down-regulation of miR-205-5p was remarkably correlated to prostate cancer and castration-resistant prostate cancer [26]. Moreover, miRNAs possess the ability to post-transcriptionally modulate gene expression by interacting with the 3′UTR of specific target mRNAs [27]. Consistently, in the present study, the biological prediction website and luciferase reporter assay identified that miR-205-5p bound to the 3′UTR of PRKCE protein and negatively regulate its expression. A study about prostatic carcinoma found that miR-205-5p inhibited cell migration and invasion by targeting zinc finger E-box binding homeobox 1 expression [28]. In consent with our study, it has been demonstrated that miR-146a can potentially bind to the 3′UTR of PRKCE [24].

Another key observation of the current study indicated that overexpression of miR-205-5p or silencing PRKCE promoted apoptosis and suppressed the drug resistance of GBC stem cells, accompanied by increased expressions of Bax and cleaved caspase 3, while decreased expression of Bcl-2. The Bax and Bcl-2 are well-known members of the Bcl-2 family of proteins [29]. While Bax is the member of pro-apoptotic Bcl-2 family of proteins and Bcl-2 is a negative regulator of cell death [11]. However, caspase-3 is considered as the main mediator of apoptotic cell death [30]. Nevertheless, the miRNAs have pivotal roles in a variety of biological processes, such as differentiation, cell growth, and cell death [31]. Concurrent with our results, it has been revealed that the ectopic expression of miR-205 subdues proliferation and tumor growth, induces apoptosis, and sensitizes pancreatic cancer cells to gemcitabine [16].

While PRKCE is a member of protein kinase C (PKC) and a promoter of gemcitabine resistance and correlates to the poor prognosis in GBC tumors [32]. According to the recently reported study, PRKCE is an anti-apoptotic gene that is often down-regulated to promote the survival of different types of cancers [33]. The down-regulation of PRKCE has been indicated to suppresses the proliferation potential, resistance to chemotherapeutics, and tumor formation ability in renal cell carcinoma *in vivo* [34]. Moreover, PRKCE promotes cancer development by acting as an anti-apoptotic gene [33]. Inconsistent to our study, the results from the tumor xenograft mouse model provided evidence that miR-218-5p enhanced gemcitabine-induced apoptosis and GBC chemosensitivity by targeting PRKCE [8]. Thus, suggesting the potential of PRKCE inhibition to attenuate GBC chemoresistance, which merits our further investigation.

## Conclusion

**Figure 6:**
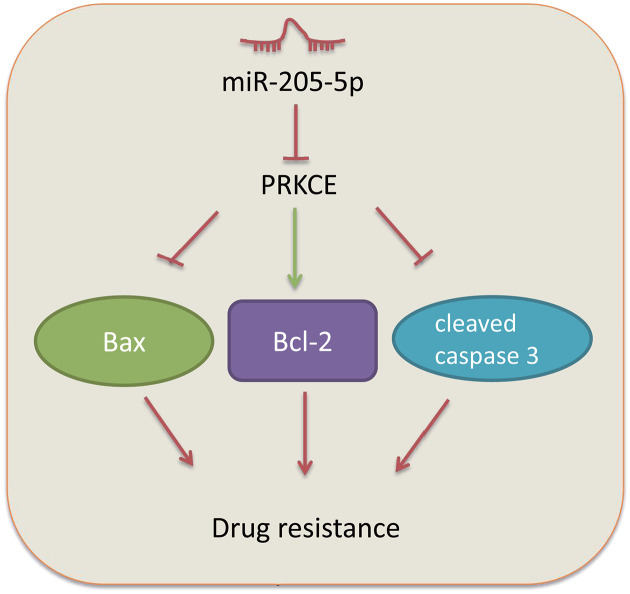
Molecular mechanisms of miR-205-5p affecting the development of GBC miR-205-5p enhances the expression of Bax and cleaved caspase 3 and inhibits the expression of Bcl-2, further decreasing the drug resistance and enhancing the apoptosis of GBC stem cells by down-regulating PRKCE.

In summary, our results suggested that miR-205-5p can promote the GBC stem cell apoptosis and further sensitize the GBC stem cells to gemcitabine by inhibiting the PRKCE expression (Figure 6). These findings enlightened that miR-205-5p could be a promising target for GBC treatment, especially for gemcitabine-treated cases. However, the underlying mechanism of gemcitabine resistance in GBC is considered as complex and multifactorial. Therefore, further investigations are required to reveal the precise molecular mechanism indicating the involvement of PRKCE in GBC.

## Supplementary Material

Supplementary Figure S1

## Abbreviations

Bax: Bcl-2-associated X protein
Bcl-2: B-cell lymphoma 2
CCK-8: cell counting kit-8
DAB: diaminobenzidine
EDTA: ethylenediaminetetraacetic acid
FITC: fluorescein isothiocyanate
GAPDH: glyceraldehyde-3-phosphate dehydrogenase
GBC: gallbladder cancer
HRP: horseradish peroxidase
miR/miRNA: microRNA
MUT: mutant type
NC: negative control
OD: optical density
OS: overall survival
PI: propidium iodide
PRKCE: protein kinase C ϵ
RLU: relative luciferase
RT-qPCR: reverse transcription-quantitative polymerase chain reaction
TUNEL: terminal deoxynucleotidyl transferase dUTP nick end-labeling
UTR: untranslated region
WT: wildtype
