# Supplementary material for: Overexpression of microRNA-205-5p exerts suppressive effects on stem cell drug resistance in gallbladder cancer by down-regulating PRKCE
Source: Biosci Rep. 2020 Sep 29;40(9):BSR20194509. doi: 10.1042/BSR20194509 (PMC7533283; doi:10.1042/BSR20194509)
Supplement: Supplementary Figure S1 [file BSR-2019-4509_supp.pdf]

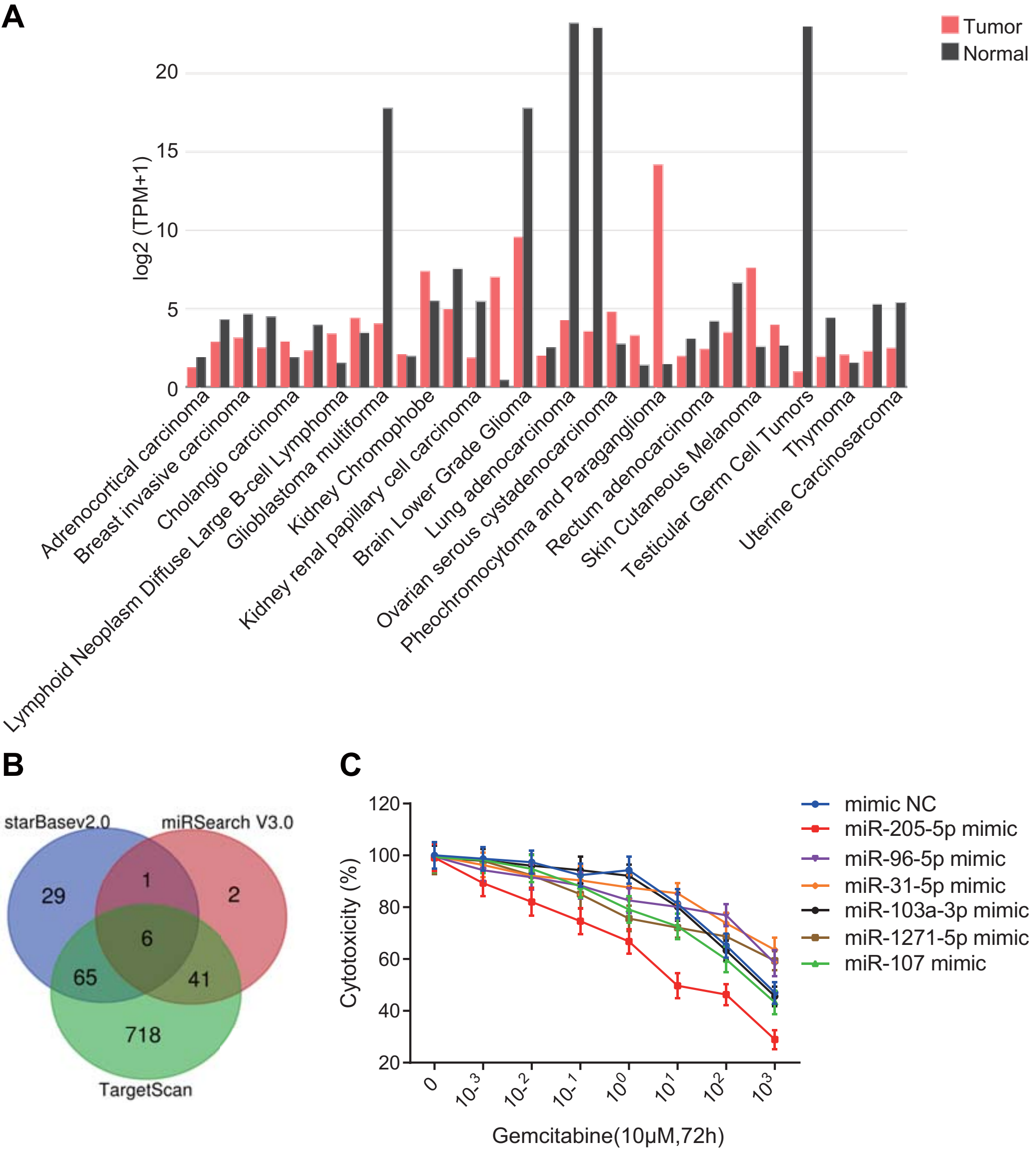
A
Tumor
Normal
log2 (TPM+1)
0
5
10
15
20
Adrenocortical carcinoma
Breast invasive carcinoma
Cholangio carcinoma
Lymphoid Neoplasm Diffuse Large B-cell Lymphoma
Glioblastoma multiforma
Kidney Chromophobe
Kidney renal papillary cell carcinoma
Brain Lower Grade Glioma
Lung adenocarcinoma
Ovarian serous cystadenocarcinoma
Pheochromocytoma and Paraganglioma
Rectum adenocarcinoma
Skin Cutaneous Melanoma
Testicular Germ Cell Tumors
Thymoma
Uterine Carcinosarcoma
B
starBasev2.0
miRSearch V3.0
TargetScan
29
1
2
6
65
41
718
C
Cytotoxicity (%)
20
40
60
80
100
120
0
10-3
10-2
10-1
100
101
102
103
Gemcitabine(10μM,72h)
mimic NC
miR-205-5p mimic
miR-96-5p mimic
miR-31-5p mimic
miR-103a-3p mimic
miR-1271-5p mimic
miR-107 mimic

Supplementary Figure 1 miRNA and mRNA expression profiles in GBC stem cells. A, TCGA data analysis of PRKCE expression in different cancers. Abscissa represents disease name, and ordinate represents expression value (log2 [TPM + 1]). B, Venn map of the predicted miRNAs targeting PRKCE. C, cytotoxicity of cells after overexpression of miRNAs determined by CCK-8 assay. ACC, adrenocortical carcinoma; BRCA, breast invasive carcinoma; CHOL, cholangiocarcinoma; DLBC, lymphoid neoplasm diffuse large b-cell lymphoma; GBM, glioblastoma multiforme; HNSC, head, and neck squamous cell carcinoma; KICH, kidney chromophobe; LGG, brain lower grade glioma; LUAD, lung adenocarcinoma; OV, ovarian serous cystadenocarcinoma; PCPG, pheochromocytoma, and paraganglioma; READ, rectum adenocarcinoma; SARC, sarcoma; STAD, stomach adenocarcinoma; TGCT, testicular germ cell tumors; THYM, thymoma; UCS, uterine carcinosarcoma.
